# Development and validation of a machine learning-derived radiomics model for diagnosis of osteoporosis and osteopenia using quantitative computed tomography

**DOI:** 10.1186/s12880-022-00868-5

**Published:** 2022-08-08

**Authors:** Qianrong Xie, Yue Chen, Yimei Hu, Fanwei Zeng, Pingxi Wang, Lin Xu, Jianhong Wu, Jie Li, Jing Zhu, Ming Xiang, Fanxin Zeng

**Affiliations:** 1 Department of Clinical Research Center, Dazhou Central Hospital, No.56 Nanyuemiao Street, Tongchuan District, Dazhou, 635000 Sichuan China; 2 Department of Laboratory Medicine, The Third People’s Hospital of Chengdu, Chengdu, 610000 China; 3 Department of Clinical Medicine, Chengdu University of Traditional Chinese Medicine, No. 37 Shi-er-qiao Road, Jinniu District, Chengdu, 610000 Sichuan China; 4 Department of Orthopedics, Hospital of Chengdu University of Traditional Chinese Medicine, Chengdu, 610000 China; 5 Department of Bone Disease, Dazhou Central Hospital, Dazhou, 635000 China; 6 Department of Medical Imaging, Dazhou Central Hospital, Dazhou, 635000 China; 7 Department of Rheumatology and Immunology, Sichuan Academy of Medical Sciences and Sichuan Provincial People’s Hospital, No.32 First Ring Road West, Jinniu District, Chengdu, 610000 Sichuan China; 8 Department of Orthopedics, Sichuan Provincial Orthopedic Hospital, Chengdu, 610000 China

**Keywords:** Combined clinical-radiomic model, Osteoporosis, Osteopenia, Quantitative computed tomography

## Abstract

**Background:**

To develop and validate a quantitative computed tomography (QCT) based radiomics model for discriminating osteoporosis and osteopenia.

**Methods:**

A total of 635 patients underwent QCT were retrospectively included from November 2016 to November 2019. The patients with osteopenia or osteoporosis (N = 590) were divided into a training cohort (N = 414) and a test cohort (N = 176). Radiomics features were extracted from the QCT images of the third lumbar vertebra. Minimum redundancy and maximum relevance and least absolute shrinkage and selection operator were used for data dimensional reduction, features selection and radiomics model building. Multivariable logistic regression was applied to construct the combined clinical-radiomic model that incorporated radiomics signatures and clinical characteristics. The performance of the combined clinical-radiomic model was evaluated by the area under the curve of receiver operator characteristic curve (ROC–AUC), accuracy, specificity, sensitivity, positive predictive value, and negative predictive value.

**Results:**

The patients with osteopenia or osteoporosis were randomly divided into training and test cohort with a ratio of 7:3. Six more predictive radiomics signatures, age, alkaline phosphatase and homocysteine were selected to construct the combined clinical-radiomic model for diagnosis of osteoporosis and osteopenia. The AUC of the combined clinical-radiomic model was 0.96 (95% confidence interval (CI), 0.95 to 0.98) in the training cohort and 0.96 (95% CI 0.92 to 1.00) in the test cohort, which were superior to the clinical model alone (training-AUC = 0.81, test-AUC = 0.79). The calibration curve demonstrated that the radiomics nomogram had good agreement between prediction and observation and decision curve analysis confirmed clinically useful.

**Conclusions:**

The combined clinical-radiomic model that incorporates the radiomics score and clinical risk factors, can serve as a reliable and powerful tool for discriminating osteoporosis and osteopenia.

**Supplementary Information:**

The online version contains supplementary material available at 10.1186/s12880-022-00868-5.

## Introduction

Osteoporosis is a age-related musculoskeletal disease characterized by reduced bone mass and increasing of bone fragility and fracture susceptibility [1]. Obviously, aging of population is becoming more severe and it has been estimated about 6 million fragility fractures will occur in China by 2050 [2]. Early screening and intervention will effectively slow down the development of bone resorption and reduce the risk for initial or subsequent fractures [3].

Dual energy X-ray (DXA) is currently widely used for diagnosis of osteoporosis, but it may be interfered by vascular calcification, osteophyte, and body position. Quantitative computed tomography (QCT) is also an imaging technique based on radiation absorption to measure volumetric density, which can assess cortical and trabecular bone compartments separately [4]. The changes of bone mineral density (BMD)measured by QCT are more sensitive to age-related or treatment-related than that measured by DXA for the whole vertebral body [4].

Since radiomics [5–9] was proposed in 2012, a high-throughput approach of mining-specific image characteristics from standard medical images, which has drawn increasing attentions. It extracts a large number of features and applies them to the clinical decision support systems to improve the accuracy of qualitative evaluation and prognosis of lesions. Radiomics has been successfully applied in prediction and differentiation of disease outcome in high-risk prostate cancer [10], bone mineral loss [11], osteopenia and osteoporosis for X-ray [12], osteoporosis prediction [13] and types of multiple myeloma [5]. Tagliafico AS et al. [5] was the first study bringing radiomics signatures into musculoskeletal system to differentiate bone tissue into focal and diffuse pattern of multiple myeloma, and their model validation effective rates were 73–71%, respectively.

Therefore, our study aimed to establish and validate a nomogram radiomics model that incorporated both the radiomics signatures based on QCT images and clinic risk factors to evaluate osteoporosis and osteopenia for individual pre-treatment.

## Methods

### Patients

**Fig. 1:**
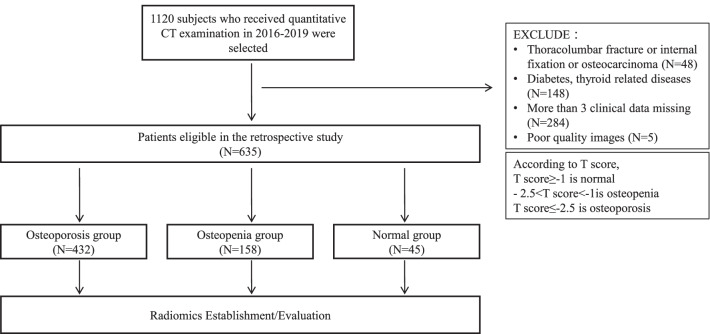
Flowchart of this study

The participants were included from Dazhou Central Hospital between November 2016 and November 2019. 1120 cases who received QCT were retrospectively collected (Fig. 1). The exclusion criteria were as followed: i. lumbar fracture or lumbar fracture with internal fixation; ii. malignant space occupying lesions of lumbar vertebra; iii. metabolic or endocrine diseases such as hyperthyroidism or hypothyroidism, occupying lesions of thyroid, diabetes, neurological diseases (such as parkinson’s disease and alzheimer’s disease); iv. chronic obstructive pulmonary disease; v. poor quality images; vi. more than 3 items of clinical characteristics were missed. Gender, age, hemoglobin (Hb), glucose, total bilirubin, direct bilirubin, indirect bilirubin, alkaline phosphatase (ALP), uric acid (UA), calcium (Ca), magnesium (Mg), phosphorus (P), homocysteine (Hcy) and other clinical information in the same period were collected from the medical records of patients. The patients were classified into normal (T-score ≥ − 1), osteopenia (− 2.5 < T-score < − 1) and osteoporosis (T-score ≤ − 2.5) groups according to the T-score. The T-score was calculated from bone mineral density. The calculation formula is: Db = (Hb − Hw)/(Hk − Hw) × Ck, T-score = (BMD_(Patients)_ − BMD_(Healthy young people)_)/SD_(Control)_, which Db was the BMD, Hb was the CT value of ROI, Hk was the CT value ofphantom, Hw was the CT value of water, and the Ck was the density of phantom. The study was approved by the ethics committee of Dazhou Central Hospital and kept with the policies for a retrospective review, informed consent was not required.

### Region of interest (ROI) segmentation and feature extraction

The images were acquired using SOMATOM Definition AS (Siemens Healthcare, Forchheim, Germany). The QCT scan parameters were 80 kV tube voltage, 10 mm slice width, 1 s exam time. The T-score were obtained by analyzing QCT images with osteo software (Siemens Healthcare, Forchheim, Germany). The image parameters were as follows: acquisition matrix = 512 × 512. The CT images were retrieved from the INFINITT Picture Archiving and Communication Systems (PACS) (INFINITT Healthcare, Seoul, Korea)and then exported to the 3D slicer software (version 4.10.2; www.slicer.org) (USA National Institutes of health, Bethesda, USA)for manual segmentation. In this study, all the images were Digital Imaging and Communications in Medicine (DICOM) format. The ROI were manually segmented by two medical students under the guidance of experienced radiologists. Pyradiomics installed in 3D slicer was used to extract feature [14] and the wavelet filters was applied in the feature extraction steps, which is compliant with Image Biomarker Standardization Initiative (IBSI) [15]. We did not perform voxel resampling. The initial setting used for the feature extraction process was showed in Additional file 1: Table S1. The cancellous bone of the third lumbar vertebrae (L3) were segmented and 851 quantitative features were produced. These features included shape, gray level dependence matrix (gldm), gray-level co-occurrence matrix (glcm), firstorder, gray-level run-length matrix (glrlm), gray-level size zone matrix (glszm), neighborhood gray-tone difference matrix (ngtdm).

### Inter- and Intra-observer Reproducibility Evaluation

Twenty consecutive patient images were selected for inter- and intra- observer reproducibility comparison. Each observer repeated the generation 851 of radiomic features twice within a half year period following the same procedure to assess intra-observer reproducibility.

### Radiomics nomogram construction

**Fig. 2:**
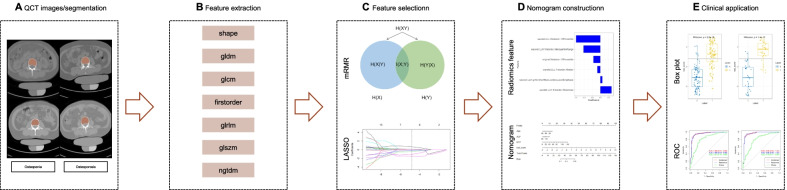
Flowchart of the radiomics method

Eligible patients were randomly allocated to training and test cohorts in a ratio of 7:3. The “preProcess” function in R was used for feature standardization (centering and scaling). Minimum redundancy and maximum relevance (mRMR) [16] was used to pre-selected 851 radiomics signatures, then the least absolute shrinkage and selection operator (LASSO) was further selected the remained features to reduce redundancy. The mRMR utilizes the information entropy and difference to select features, so that the selected features have the minimum redundancy and meet the maximum correlation. LASSO regression model excludes the secondary independent variable coefficient by regression penalty. Multiple logistic regression model was used to select candidate factors of clinical information, such as age, gender, and other biochemistry metrics. An overview of the combined model process was shown in the Fig. 2.

### Statistical analysis

Statistical analysis was performed using SPSS 20.0 (International Business Machines Corporation, State of New York, USA) and R software (version 3.6.1) (R studio, Boston, USA). The “mRMRe” and “glmnet” packages in R were used to build mRMR and LASSO. The nomogram was built using “rms” package. The independent sample *t*-test or Mann–Whitney *U* test was used to analyze the relationship between quantitative data (Age, HGB, GLU, TBIL, DBIL, IBIL, ALP, UA, Ca, Mg, P, HCY), but the Chi-square test was used to analyze the categorical data (Gender). The area under the curve (AUC), accuracy, specificity, sensitivity, positive predictive value and negative predictive value were used to evaluate the performance of the model for distinguishing the osteoporosis and osteopenia. In order to assess the difference of receive operating characteristic, the Delong’s test was used. The calibration curves accompanied by Hosmer–Lemeshow *H* test were used to evaluate whether the model was perfectly calibrated. Intra-class correlation coefficients (ICCs) was used to evaluated the inter- and intra- observer reproducibility based the extracted features, which 0.81–1.00 was considered to be perfect agreement. Two-tailed *P* < 0.05 was considered to have significant difference.

## Results

### Patients

A total of 635 patients were eligible for inclusion in our study (Fig. 1). We divided patients into a training cohort (N = 414) and a test cohort (N = 176). The characteristics of patients were presented in Table 1 and Additional file 1: Table S1. Age, ALP and Hcy were the top three different clinical characteristics between osteoporosis and osteopenia in the training group (*P* < 0.05).

**Table 1:** Characteristics of Patients in the Training and Test Cohorts

Characteristics	Training cohorts			Test cohorts		*P*
	Osteoporosis	Osteopenia	*P*	Osteoporosis	Osteopenia	
Gender, No. (%)			0.032*			0.897
Male	86 (28.40)	20 (18.00)		29 (22.50)	11 (23.40)	
Female	217 (71.60)	91 (82.00)		100 (77.50)	36 (76.60)	
Age, mean (SE), years	70.03 (0.55)	58.32 (0.92)	< 0.001*	69.11 (0.85)	57.72 (1.45)	< 0.001*
HGB, mean (SE), g/L	122.28 (1.67)	127.83 (1.59)	0.209	125.46 (1.54)	127.17 (2.60)	0.385
GLU, mean (SE), mmol/L	5.56 (0.07)	5.21 (0.13)	0.010*	5.80 (0.15)	5.28 (0.08)	0.010*
TBIL, mean (SE), umol/L	17.18 (3.83)	13.02 (0.59)	0.354	14.52 (0.69)	14.86 (0.89)	0.341
DBIL, mean (SE), umol/L	4.66 (0.91)	3.11 (0.13)	0.001	4.30 (0.35)	3.74 (0.34)	0.435
IBIL, mean (SE), umol/L	13.56 (3.95)	9.91 (0.49)	0.935	10.22 (0.45)	11.17 (0.70)	0.115
ALP, mean (SE), U/L	84.49 (1.50)	76.13 (2.40)	0.001*	85.06 (3.36)	75.84 (3.64)	0.153
UA, mean (SE), umol/L	301.77 (4.69)	305.54 (8.48)	0.851	307.97 (8.39)	296.66 (10.19)	0.351
Ca, mean (SE), mmol/L	2.64 (0.34)	2.34 (0.01)	0.001	2.29 (0.01)	2.33 (0.02)	0.049*
Mg, mean (SE), mmol/L	1.06 (0.01)	1.04 (0.02)	0.968	1.07 (0.02)	1.01 (0.02)	0.062
P, mean (SE), mmol/L	1.09 (0.01)	1.13 (0.02)	0.047*	1.09 (0.01)	1.10 (0.02)	0.858
HCY, mean (SE), umol/L	14.83 (0.58)	11.48 (0.50)	< 0.001*	14.90 (0.86)	12.83 (0.71)	0.252

*P* value is derived from the univariable association analyses. Chi-Square was used to analyze the difference of categorical data (Gender), while the independent sample *t*-test or Mann–Whitney *U* test was used to analyze the difference of quantitative data (Age, HGB, GLU, TBIL, DBIL, IBIL, ALP, UA, Ca, Mg, P, HCY)

*HGB* hemoglobin, *GLU* glucose, *TBIL* total bilirubin, *DBIL* direct bilirubin, *IBIL* indirect bilirubin, *ALP* alkaline phosphatase, *UA* uric acid, *Ca* calcium, *Mg* magnesium, *P* phosphorus, *HCY* homocysteine, *SE* standard error

^*^P value < 0.05

### Feature selection and radiomics score (rad-score) construction

Supporting Information (ICCs of intra- and inter- observer was perfect agreement.)

A combined mRMR and LASSO methodological approach was used to select the 6 optimal radiomics features. Firstly, there were 20 potential predictors from 851 features selected by the mRMR (Additional file 1: Fig. S1A). Then, the LASSO was further selected 6 optimal features with nonzero coefficients (Additional file 1: Fig. S1B, C). The features included wavelet_LLL_firstorder_10Percentile, wavelet_LLH_firstorder_InterquartileRange, original_firstorder_10Percentile, wavelet_LLL_firstorder_Median, wavelet_LLH_glrlm_ShortRunLowGrayLevelEmphasis, wavelet_LLH_firstorder_Skewness. The contribution of the selected features was shown in Additional file 1: Fig. S2 and the rad-score were shown in supplementary materials. The rad-score showed the excellent diagnosis performance in discriminating the osteoporosis and osteopenia in training (*P* < 0.001) and test cohort (*P* < 0.001) (Additional file 1: Fig. S3).

### Development and validation of a radiomics model

**Fig. 3:**
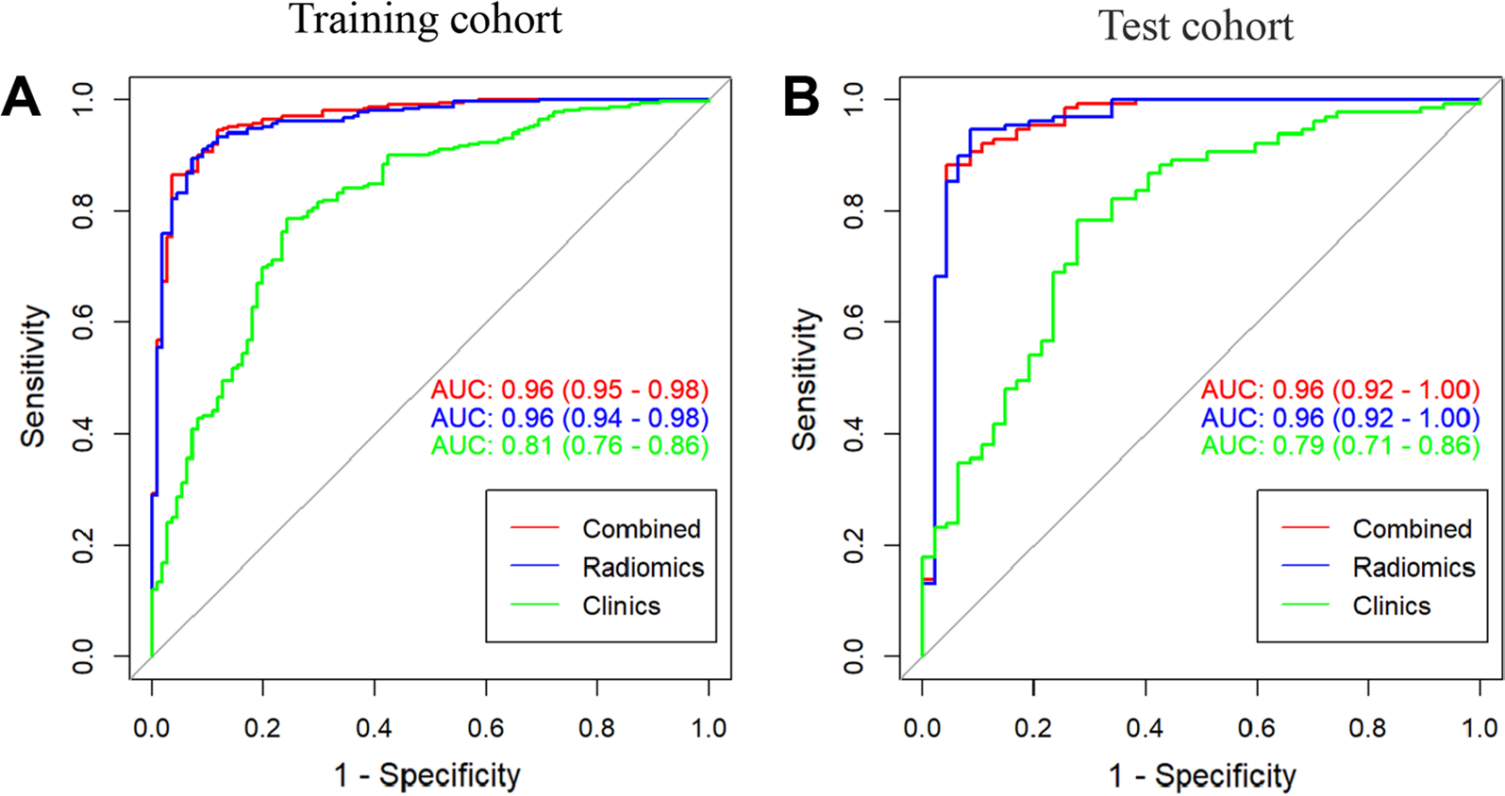
Receive operating characteristic curve (ROC) for the radiomics model, clinical model and combined model in distinguishing osteoporosis and osteopenia. **A** The model comparison in the training cohort. **B** The model comparison in the test cohort

**Fig. 4:**
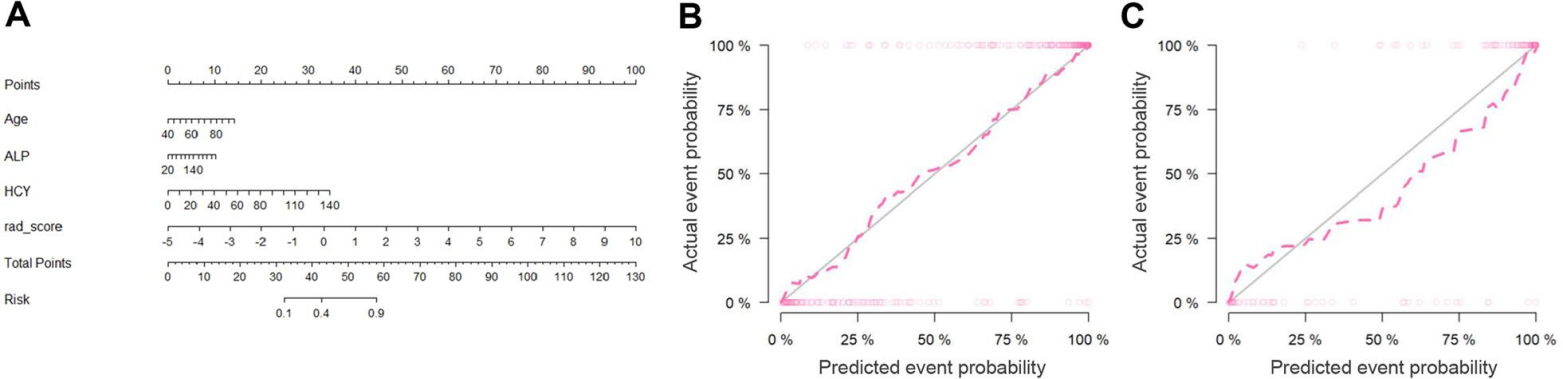
Development and performance of nomogram. **A** Nomogram based on radiomics signatures and clinical factors. **B** Calibration curves of the radiomics nomogram in the training cohort. **C** Calibration curves of the radiomics nomogram in the test cohorts

We constructed two models to distinguishing osteoporosis and osteopenia based on the radiomics features or clinical variables, respectively. The AUC of the radiomics model was 0.96 (95% confidence interval (CI), 0.94–0.98) in the training cohort and 0.96 (95% CI 0.92–1.00) in the test cohort (Fig. 3). The clinical model yielded an AUC of 0.81 (95% CI 0.78–0.86) in the training cohort and 0.79 (95% CI 0.71–0.86) in the test cohort. Furthermore, the model combined with the clinical variables and radiomics features of AUC was 0.96 (95% CI 0.95–0.98) in the training cohort, while the AUC was 0.96 (95% CI 0.92–1.00) in the test cohort. The results showed that the radiomics model was not inferior to the combined clinical-radiomic model both in the training and test cohort (Fig. 3). In addition, we compared the diagnostic performance among the clinics, radiomics and combined model using accuracy, sensitivity, specificity both in the training and test group (Table 2). A nomogram was conducted based on the rad-score and 3 clinical characteristics (Age, ALP, Hcy) for differentiation of osteoporosis and osteopenia (Fig. 4A). The calibration curve showed good agreement between prediction and observation in both cohort (Fig. 4B, C). The *P*-value (0.95 in the training cohort and 0.09 in the test cohort) using the Hosmer–Lemeshow *H* test showed had no departure from the perfect fit. Furthermore, we compared the normal and patients with bone loss to validate the performance of the rad-score in different subgroups. The results were considerably good especially in differentiating osteoporosis and normal subjects (AUC = 0.99, 95% CI 0.98–1.00), and the AUC of bone loss patients and normal subjects was 0.90 (95% CI 0.87–0.93), the AUC of osteopenia and normal was 0.66 (95% CI 0.57–0.75) (Additional file 1: Fig. S4).

**Table 2:** Diagnostic performance of clinical, radiomics and combined clinical-radiomic model

Group	Model	Accuracy	Sensitivity	Specificity	PPV	NPV
Training	Clinics	0.78	0.90	0.56	0.79	0.76
Test	Clinics	0.74	0.88	0.51	0.74	0.72
Training	Radiomics	0.90	0.93	0.89	0.76	0.97
Test	Radiomics	0.94	0.92	0.95	0.86	0.97
Training	Combined clinical-radiomics	0.89	0.99	0.72	0.87	0.96
Test	Combined clinical-radiomics	0.90	0.98	0.75	0.88	0.96

*PPV* positive predictive value, *NPV* negative predictive value

### Clinical use

**Fig. 5:**
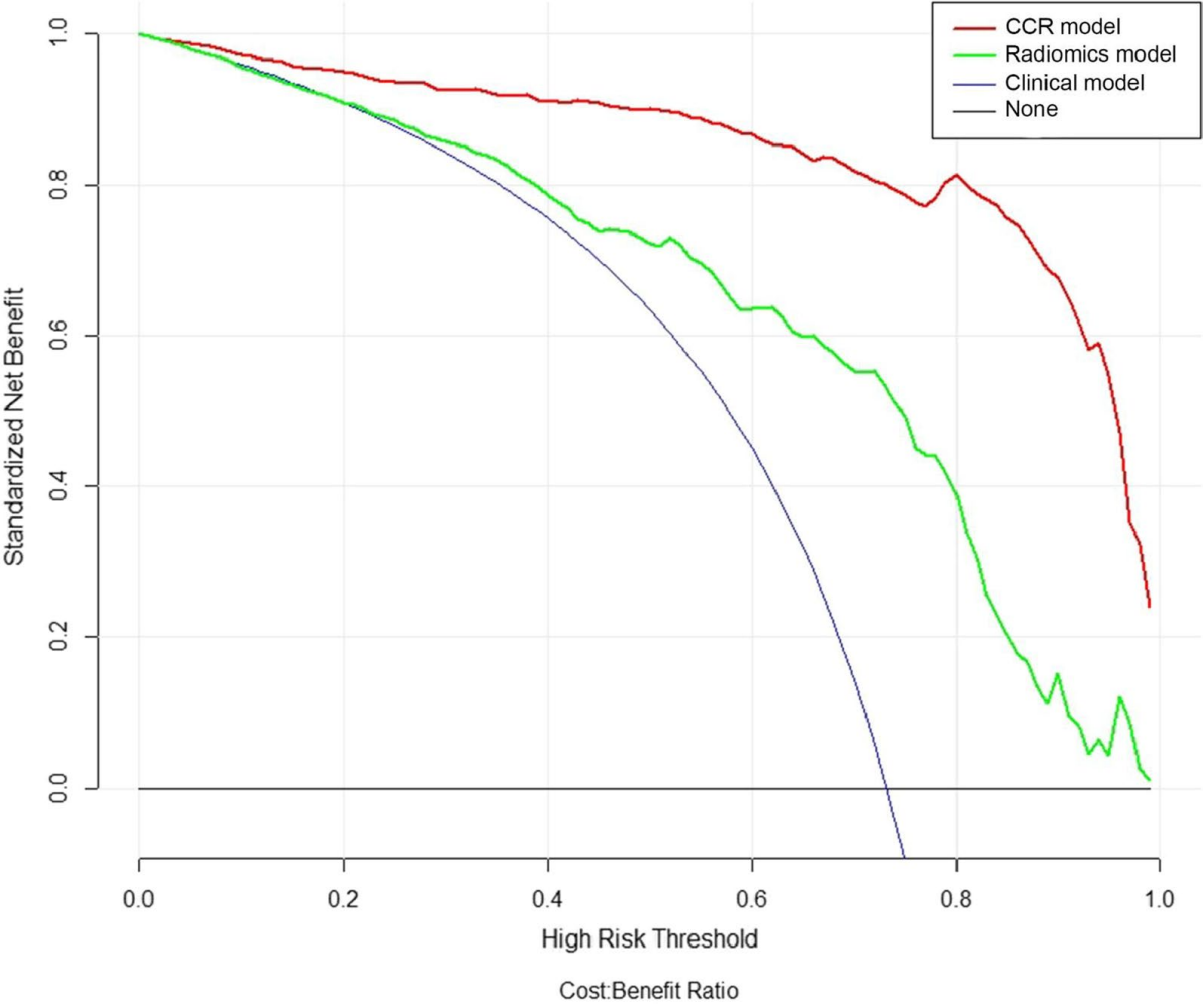
Decision curve analysis for radiomics nomogram and signature

The decision curve of the radiomics, clinical and combined clinical-radiomic model was shown in Fig. 5. The nomogram with rad-score was considered better than clinical model in discriminating the osteoporosis and osteopenia when the threshold probability in the early stage. The net benefit of combined clinical-radiomic model was higher than clinical model in all the range.

## Discussion

This study built and validated a diagnostic model of bone mass individualization based on QCT images, with a large sample that successfully distinguished the osteoporosis and osteopenia patients. We found that the radiomics features can supplement the current clinical diagnostic system, and it provides a better discrimination and evaluation ability for the development of bone loss meanwhile.

BMD is the most important parameter in the diagnosis of osteoporosis. However, it can not reflect the bone microstructure. In comparison to BMD obtained by QCT, bone microstructure parameters brings additional and complementary information to improve the prediction of fracture risk [17, 18]. As a new technology, radiomics can produce many features that reflect the microstructure of images. Some studies have shown that there is a certain correlation between the texture parameters of cancellous bone extracted from radiological images and bone microstructure [19, 20]. He et al. have established a radiomics model based on MRI for the diagnosis of osteoporosis [21]. In our study, the model based on QCT images and clinical data can quantify the information of higher dimensions of vertebral body to a certain extent, which is comprehensively analyze the changes of bone structure and bone mineral density, and has certain application value for the evaluation of osteoporosis.

For the construction of the radiomics signatures, 851 candidate radiomics features were shrunk to 20 potential predictors with mRMR feature selection to eliminate the redundant and irrelevant features. Then, LASSO approach was used to reduce the regression coefficient, extract 6 features with high performance of discrimination for osteoporosis and osteopenia to establish rad-score. Signatures extracted from LASSO were considerable accurately and the regression coefficients of most signatures were reduced toward zero during overfitting [22], making the model easier to construe and available to identify the top ranking signatures [23]. Compared to the clinical predictors alone (training-AUC = 0.81, test-AUC = 0.79), the combined model showed significant improvement (AUC = 0.96).

We used QCT images to extract the meaningful radiomics features in discriminating bone density changes and osteoporosis and received a considerable performance (AUC = 0.96). Previous model [24] based on DXA images investigate the diagnostic accuracy of bone mass classification using several machine learning algorithms, showing a small sample capacity and a lower AUC that ranging between 0.50 and 0.78. Lee KS et al. explored the reliability the reliability of using dental tablets to screen osteoporosis (AUC = 0.858) [25]. Several studies has confirmed that [26–28] the lumbar spine is the good observation site for bone loss in all skeletal structures, among long bone backbone, femoral neck, and spinal segment [29, 30]. Among the lumbar vertebrae, the L3 has the highest specificity [26], because L3 is found to be the vertebral body with the highest bone metabolism rate and the lowest bone density in all vertebral bodies. In our study, the L3 was used to extract radiomics signatures, and it was proved that the extracted signatures could significantly discriminate the osteoporosis and osteopenia (AUC = 0.96). In comparison, some studies developed a predictive model for BMD based on hip BMD [31], lumbar and hip BMD [32] or unspecified [33]. The delineation of ROI is the most important part in the analysis of radiomics, because the subsequent data is generated by the delineated area, and the gray scale of cancellous bone and cortical bone is clear and easy to distinguish.

The 6 more predictive radiomics signatures mainly concluded wavelet_LLH features, which had been proved having high discriminating and predictable performance of model verification, especially in distant metastases of lung cancer [34] and improvement of radiomics reproducibility for pulmonary nodules or masses [35]. In these 6 features, 2 were the wavelet_LLL features, 3 were wavelet_LLH features and 1 was original feature. In addition, features include both first-order features and grayscale features. Wavelet features could reflect the spatial heterogeneity of vertebral body synthetically cause they contain high-order image [36]. Studies have shown that both skewness and glrlm can reflect the heterogeneity of regions of interest [37–39]. Our analysis demonstrated that the 6 radiomics features had a strong discrimination ability to evaluate osteoporosis and osteopenia. Previous studies [40–42] proposed that age, gender, Hb, UA, Hcy, ALP, Ca, are related with osteoporosis, but the correlations are weakly positive compared to BMD [40]. In our study, the specific clinical predictors performed a training AUC of 0.81 and a test AUC of 0.79. Therefore, our results showed that adding the rad-score to the model can improve the performance of clinical nomogram (training-AUC = 0.96, test-AUC = 0.96). This finding may support multi-dimension data is most effectively way to construct clinical decision model. However, some studies only use clinical data to diagnose osteoporosis [43, 44]. There are several limitations in our study. First, this study was derived from single center and there is no external data, so its performance evaluation may be overly optimistic. Second, the follow-up data like therapeutic response and fracture rate was not considered currently.

In summary, a rad-score derived from QCT images was proposed in this study, which was an independent risk factor for abnormal BMD patients. In addition, diagnostic nomograms combining rad-score and clinical predictors provided a convenient way to discriminate osteoporosis and osteopenia and may influence decision-making on the possible benefit of treatment.

## Supplementary Information

**Additional file 1: Figure S1.** Radiomics feature selection using minimum redundancy and maximum relevance (mRMR) and least absolute shrinkage and selection operator (LASSO). **Figure S2.** The contribution of each feature to the radiomic signature was shown by histogram. **Figure S3.** Performance of rad-score in discriminating between osteoporosis and osteopenia. **Figure S4.** Performance of rad-score in discriminating between normal and osteopenia, abnormal and osteoporosis. **Table S1.** Pyradiomics plug-in unit extraction parameters (v2.2). **Table S2.** Characteristics of Patients.

## Data Availability

The datasets used and/or analysed during the current study are available from the corresponding author on reasonable request.
