# Supplementary material for: Development and validation of a machine learning-derived radiomics model for diagnosis of osteoporosis and osteopenia using quantitative computed tomography
Source: BMC Med Imaging. 2022 Aug 8;22:140. doi: 10.1186/s12880-022-00868-5 (PMC9358842; doi:10.1186/s12880-022-00868-5)
Supplement: Supplementary file 1 — Additional file 1: Figure S1. Radiomics feature selection using minimum redundancy and maximum relevance (mRMR) and least absolute shrinkage and selection operator (LASSO). Figure S2. The contribution of each feature to the radiomic signature was shown by histogram. Figure S3. Performance of rad-score in discriminating between osteoporosis and osteopenia. Figure S4. Performance of rad-score in discriminating between normal and osteopenia, abnormal and osteoporosis. Table S1. Pyradiomics plug-in unit extraction parameters (v2.2). Table S2. Characteristics of Patients. [file 12880_2022_868_MOESM1_ESM.pdf]

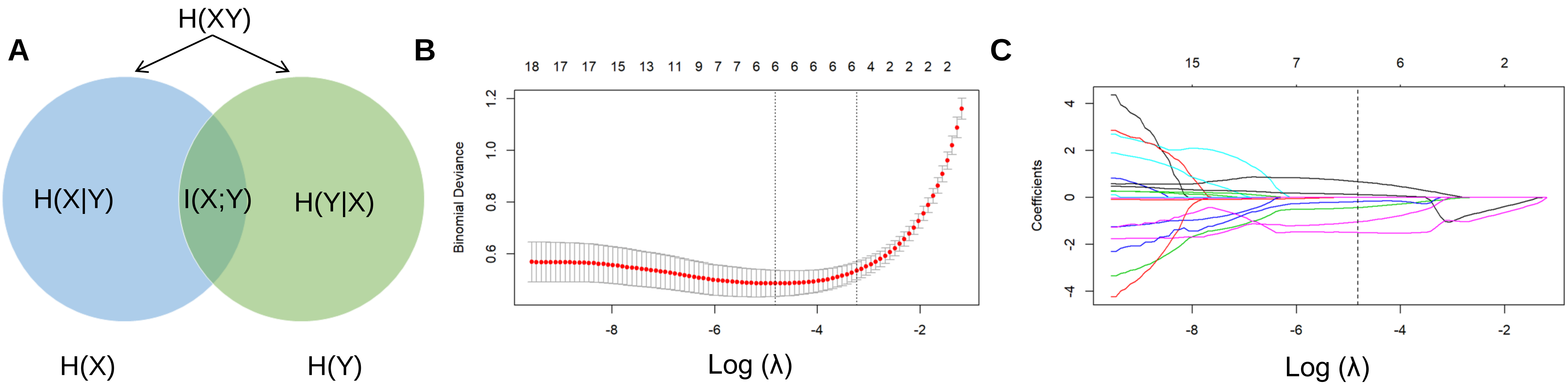

Supplementary Figure 1. Radiomics feature selection using minimum redundancy and maximum relevance (mRMR) and least absolute shrinkage and selection operator (LASSO). (**A**) mRMR was performed to eliminate the redundant and irrelevant features. (**B**) The area under the curve was plotted versus Log (λ). (**C**) LASSO coefficient profiles of the 20 texture features. A coefficient profile plot was produced against the Log (λ) sequence. Vertical line was drawn at the value selected using 10-fold cross-validation, where optimal λ resulted in 6 nonzero coefficients.

Supplementary Table 1. Pyradiomics plug-in unit extraction parameters (v2.2).

**Parameters**	Value
distances	[1]
force2Ddimension	0
force2D	False
interpolator	sitkBSpline
resampledPixelSpacing	None
normalizeScale	1
normalize	False
padDistance	5
removeOutliers	None
minimumROISize	None
binWidth	25
label	1
preCrop	False
resegmentRange	None
minimumROIDimensions	2
symmetricalGLCM	True
correctMask	True
additionalInfo	True

Supplementary Table 2. Characteristics of Patients.

**Characteristics**	Training (N=414)	Test (N=176)	*P*
Gender, No. (%)			
Male	106 (25.60)	40 (22.70)	0.459
Female	308 (74.40)	136 (77.30)	
Age, mean (SE), years	66.89 (0.54)	66.07 (0.83)	0.402
HGB, mean (SE), g/L	123.77 (1.30)	123.77 (1.79)	0.654
GLU, mean (SE), mmol/L	5.46 (0.07)	5.57 (0.12)	0.106
TBIL, mean (SE), umol/L	16.06 (2.81)	14.61 (0.56)	0.038
DBIL, mean (SE), umol/L	4.24 (0.67)	4.15 (0.27)	0.164
IBIL, mean (SE), umol/L	12.58 (2.89)	10.47 (0.38)	0.050
ALP, mean (SE), U/L	82.24 (1.28)	82.60 (2.66)	0.197
UA, mean (SE), umol/L	302.78 (4.11)	304.95 (6.72)	0.416
Ca, mean (SE), mmol/L	2.56 (0.25)	2.30 (0.01)	0.672
Mg, mean (SE), mmol/L	1.05 (0.01)	1.05 (0.01)	0.312
P, mean (SE), mmol/L	1.10 (0.01)	1.09 (0.01)	0.461
HCY, mean (SE), umol/L	13.93 (0.45)	13.95 (0.67)	0.539

NOTE. *P* value is derived from the univariable association analyses. Chi-Square was used to analyze the difference of categorical data (Age), while the independent sample *t*-test or Mann-Whitney *U* test was used to analyze the difference of quantitative data (Age, HGB, GLU, TBIL, DBIL, IBIL, ALP, UA, Ca, Mg, P, HCY)

Abbreviations: HGB, hemoglobin; GLU, glucose; TBIL, total bilirubin; DBIL, direct bilirubin; IBIL, indirect bilirubin; ALP, alkaline phosphatase; UA, uric acid; Ca, calcium; Mg, magnesium; P, phosphorus; HCY, homocysteine; SE, standard error.

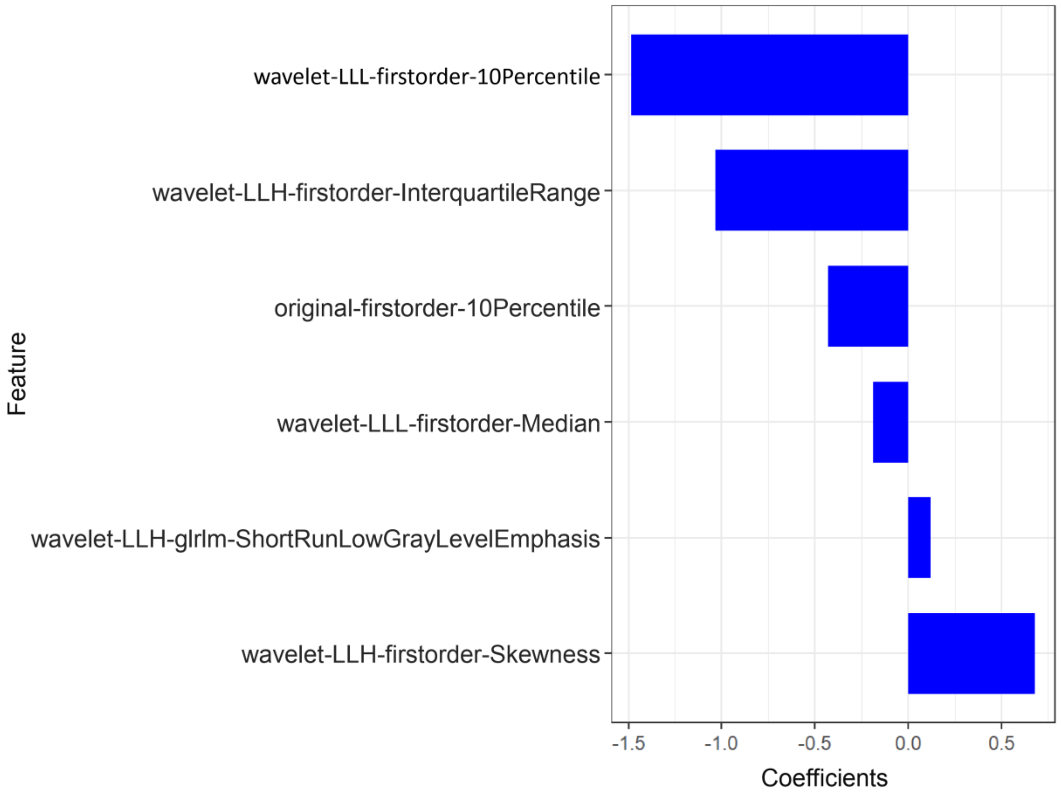

Supplementary Figure 2. The contribution of each feature to the radiomic signature was shown by histogram.

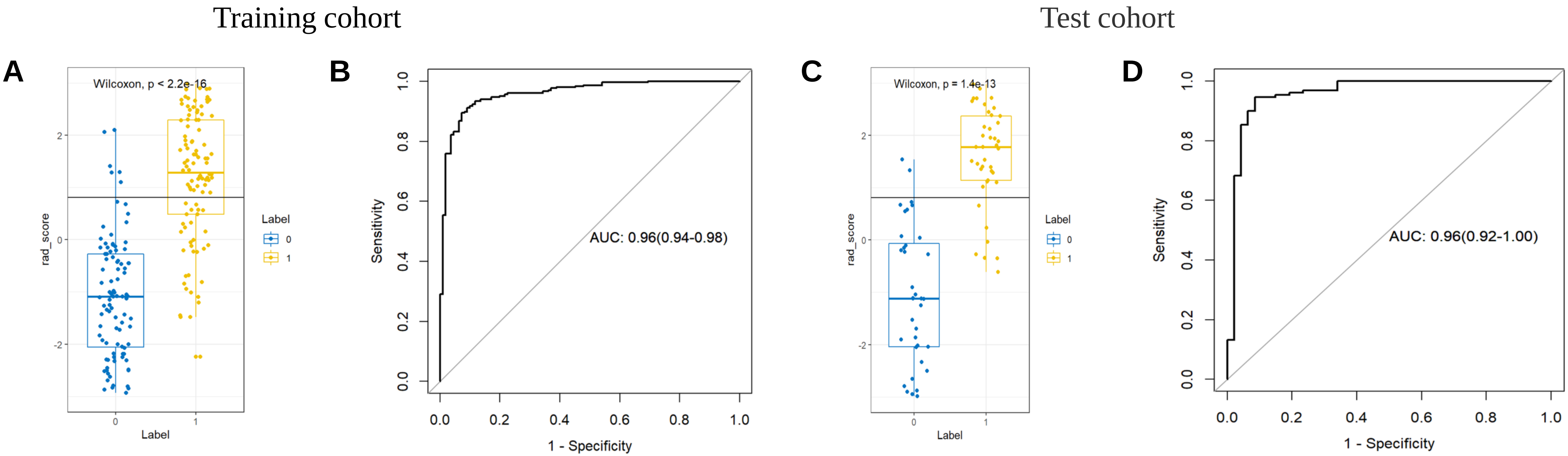

Supplementary Figure 3. Performance of rad-score in discriminating between osteoporosis and osteopenia. The box plot (**A**) and ROC (**B**) of rad-score in discriminating the osteoporosis and osteopenia in training cohort. The box plot (**C**) and ROC (**D**) of rad-score in discriminating the osteoporosis and osteopenia in test cohort. 0, osteopenia; 1, osteoporosis. Wilcoxon test, *P*<0.05. Rad, radiomics.

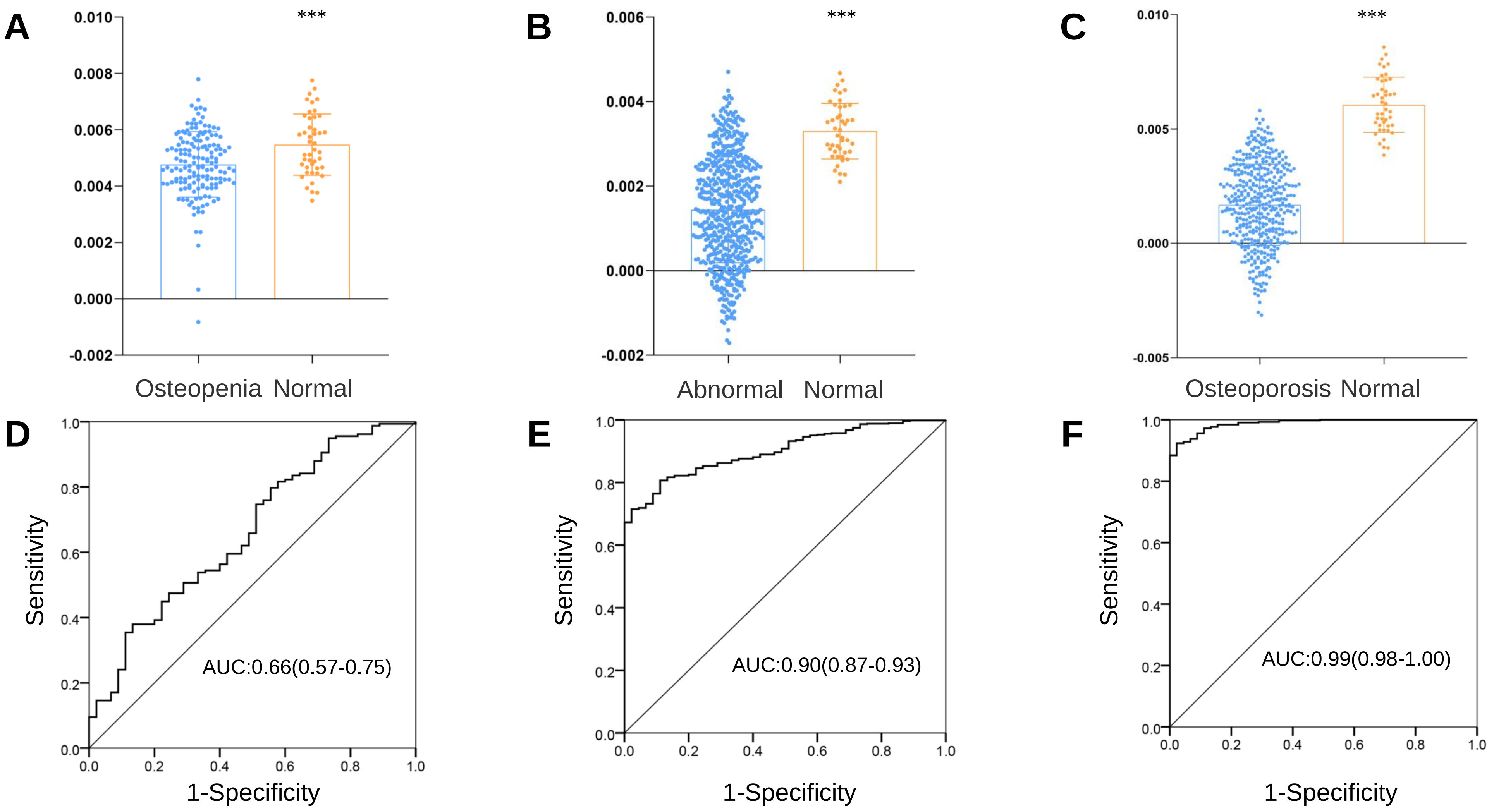

Supplementary Figure 4. Performance of rad-score in discriminating between normal and osteopenia, abnormal and osteoporosis. (**A-C**) Box plot of rad-score in discriminating between normal and osteopenia, abnormal and osteoporosis. (**D-F**) ROC of rad-score in discriminating between normal and osteopenia, abnormal and osteoporosis. Wilcoxon test, ****P* value<0.001. Osteopenia-N=158, Abnormal-N=590, Osteoporosis-N=432, Normal-N=45

Rad-score = -1.486*wavelet-LLL-firstorder-10Percentile+-1.032*wavelet-LLH-firstorder-InterquartileRange+-0.428*original-firstorder-10Percentile+-0.187*wavelet-LLL-firstorder-Median+0.123*wavelet-LLH-glrlm-ShortRunLowGrayLevelEmphasis +0.68*wavelet-LLH-firstorder-Skewness+ 2.755
